# Surgical management of acute appendicitis during the European COVID-19 second wave: safe and effective

**DOI:** 10.1007/s00068-022-02149-w

**Published:** 2023-01-19

**Authors:** Maximilian Peter Forssten, Lewis J. Kaplan, Matti Tolonen, Isidro Martinez-Casas, Yang Cao, Thomas N. Walsh, Gary Alan Bass, Shahin Mohseni, For the ESTES SnapAppy Group, Rebecka Ahl Hulme, Alan Biloslavo, Hayato Kurihara, Jorge Pereira, Arvid Pourlotfi, Éanna J. Ryan, Nayef Louri, Fatema Nedham, Thomas Noel Walsh, Jamal Hashem, Martin Corbally, Abeer Farhan, Hamad Al Hamad, Rawan Elhennawy, Mariam AlKooheji, Manar AlYusuf, Wissal Aknouche, Anas A. Zeidan, Yusuf S. Alsaffar, Edgar Lipping, Peep Talving, Sten Saar, Katrina Graumann, Liis Kibuspuu, Eduard Harkov, Gisele Aaltonen, Iines S. Sillman, Sami Haapanen, Hanna Lampela, Henna Sammalkorpi, Sofia Eskola, Altti Laakso, Johan Back, Ulla Kettunen, Antti M. Nummi, Anika Szwedyc, Taina Nykänen, Rolle Rantala, Elisa J. Mäkäräinen-Uhlbäck, Sanna A. Meriläinen, Heikki I. Huhta, Jukka M. J. Rintala, Kirsi E. M. Laitakari, Elina Lietzen, Paulina Salminen, Risto K. A. Rapola, Vahid Zangouri, Mohammad Y. Karami, Sedigheh Tahmasebi, Majid Akrami, Alireza Golchini, Faranak Bahrami, Sean M. Johnston, Sean T. Lim, Irele Ifijeh Ahonkhai, Eltahir Eltagani, Odhran K. Ryan, Ailbhe O’Driscoll-Collins, Aine O’Neill, Zakiya Penny, Orlaith Kelly, Carolyn Cullinane, Ian Reynolds, Helen Heneghan, Sean Martin, Des Winter, Matthew Davey, Maha Alkhattab, Aoife J. Lowery, Michael J. Kerin, Aisling M. Hogan, Martin S. Davey, Ke En Oh, Syed Mohammad Umar Kabir, Huilun Huan, Charlotte Aziz, Michael Sugrue, Jessica M. Ryan, Tara M. Connelly, Mohammad Alhazmi, Youssef Al-Mukhaizeem, Fiachra Cooke, Peter M. Neary, Arnold D. K. Hill, Michael R. Boland, Angus J. Lloyd, Frances Fallon, Eoin F. Cleere, James Toale, Patrick A. Boland, Michael Devine, Conor Keady, Sarah Hunter, M Kevin Barry, Michael E. Kelly, Aidan T. O’Dowling, Ben Creavin, Dara O. Kavanagh, Paul Neary, Paul F. Ridgway, Cathleen A. McCarrick, Jarlath Bolger, Barry Maguire, Cian Keogh, Surbhi Chawla, John Conneely, Emilie McCormack, Ben Shanahan, Nicola Raftery, Darragh Rice, Niall McInerney, Aine Stakelum, Jan Mares, Jonavan Tan, Mark Hanna, Ishwarya Balasubramanian, Christina Fleming, Guy Barsky, Gad Shaked, Simone Giudici, Martina Ceolin, Simona Mei, Francesca Mazzarella, Annalisa Zucca, Susanna Terranova, Nicolo de Manzini, Diego Visconti, Emanuele Doria, Mauro Santarelli, Giovanni Scotton, Francesca Notte, Giacomo Bertelli, Anna Malpaga, Giulia Armatura, Antonio Frena, Dario Tartaglia, Federico Coccolini, Camilla Cremonini, Enrico Cicuttin, Alessio Mazzoni, Massimo Chiarugi, Constança M. Azevedo, Filipa D. Mendes, Luis Q. Faria, Carlos Nazario, Daniela Machado, Miguel Semiao, Jorge Pereira, Carlos Casimiro, Jose Pinto, Tiago Pavão, Raquel Pereira, Bruno Barbosa, Nadia Tenreiro, Catia Ferreira, Goncalo Guidi, Daniela C. Martins, Clara Leal, Bruno B. Vieira, Luís S. Castro, Aldara Faria, Alberto Figueira, Mauro Sousa, Pedro Rodrigues, Rodrigo Roquette, Ricardo Ribeiro, Paulo Cardoso, Joana Domingues, Maria Isabel Manso, Rute Pereira, Tatiana Revez, Bogdan D. Dumbrava, Florin Turcu, Ionut Hutopila, Bogdana Banescu, Gerald Filip, Catalin Copaescu, Marcos Alba Valmorisco, Isabel Manzano Martín, Rocio Martín García de Arboleya, José Ortega Seda, Pablo Rodríguez González, Jose Antonio Becerra Toro, Enrique Rodríguez Lara, Jose Antonio González Minchón, Juan José Segura-Sampedro, Sebastián Jerí-McFarlane, Alejandro Gil-Catalán, Andrea Craus-Miguel, Laura Fernández-Vega, Xavier González-Argenté, Mercedes Estaire-Gómez, Borja Camacho Fernández-Pacheco, Rebeca Vitón-Herrero, Elisa Jimenez-Higuera, Alejandro Barbero, José M Valverde, Enrique Colás-Ruiz, Maria del Mar Escales-Oliver, Olga Claramonte-Bellmunt, Marta Castro-Suárez, Naila Pagés-Valle, José Andrés Cifuentes-Ródenas, Marta Merayo Alvarez, Jose Luis Michi Campos, Luis Alejandro García González, Beatriz Carrasco Aguilera, Jaime Iturbe Menéndez, Jose Luis Rodicio Miravalles, Carmen Rodríguez Haro, Sara Núñez O’Sullivan, Mariana García Virosta, María Hernández O’Reilly, Izaskun Balciscueta-Coltell, Javier Lorenzo-Perez, Sonia Martinez-Alcaide, Susana Martinez-Ramos, Maria Sebastian-Fuertes, Laura Gomez-Romer, Maria M. Pelloni, Aida Cristina Rahy-Martín, Andrés Felipe Yepes-Cano, Julio Reguera-Rosal, Jose A. Lopez-Ruiz, Beatriz Marenco, Marina Retamar-Gentil, Estela Romero-Vargas, Angeles Gil-Olarte, Aitor Landaluce-Olavarria, Begoña Estraviz-Mateos, Jose-Mario De Francisco-Rios, Aitor Sainz-Lete, Ane Emaldi-Abasolo, Manolo Leon-Valarezo, Claudia C. Lopes Moreira, Aintzane Lizarazu Perez, Araceli Rodriguez Gonzalez, Iñigo Augusto Ponce, Ignacio Maria Goena Iglesias, Cristina González-Prado, Guillermo Cabriada, Beatriz López, Michelle C. Otero, Nerea Muñoz -Plaza, Alberto Palomo, Fernando Mendoza-Moreno, Manuel Díez-Alonso, Francisca García-Moreno-Nisa, Belén Matías-García, Enrique Ovejero-Merino, Ana Quiroga-Valcárcel, Luis Sánchez-Guillén, Inmaculada Oller-Navarro, Álvaro Soler-Silva, Antonio Francisco Sanchís-López, Francisco Blanco-Antona, Luis Muñoz-Bellvis, Jaime López-Sánchez, Sonsoles Garrosa-Muñoz, Beatriz Barón-Salvador, Juan Manuel Nieto-Arranz, Andrea Campos-Serra, Raquel Gràcia-Roman, Anna Muñoz-Campaña, Carla Zerpa-Martin, Andrea Torrecilla-Portoles, Tessa Landa, Virginia Durán Muñoz-Cruzado, Felipe Pareja-Ciuró, Daniel Aparicio-Sánchez, Eduardo Perea del Pozo, Sandra Dios-Barbeito, Carlos García-Sánchez, Antonio Jesús García-Moriana, Victor Turrado-Rodriguez, Roser Termes-Serra, Paula Gonzalez-Atienza, Xavier Morales-Sevillano, Alba Torroella, César Ginestà, Alfredo Escartín, Ferney Gomez, Ana Pinillos, Jaume Ortega, Guillermo Lopez, Eric Gutierrez, Estela Membrilla-Fernandez, Francisco Ocho-Segarra, Ana María González-Castillo, Amalia Pelegrina-Manzano, Juan Guzmán-Ahumada, Juan Jose Sancho-Insenser, María Lourdes García-Jiménez, Laura Castro-Diez, Manuel González-Bermúdez, Mónica Torres-Díaz, Carla Madarro Pena, Angélica Blanco Rodríguez, Dhanisha Trivedi, Souheil Reda, Hans Edvardsson, Lovisa Strömmer, Eva-Corina Caragounis, Karin Sillén, Sofia Warfvinge, Fredrik Bergstedt, Philip Enström, Harald Olsson, Anders Rosemar, Nathalie Young, Agnieszka Popowicz, Johanna Lerström, Johanna Jäderbo, Folke Hammarqvist, Hanna Zacharias, Maria B. Wikström, Anna Stene Hurtsén, Haytham Bayadsi, Emma Jansson, Nils Brunstrom, Ellen B. Malers, Per I. Loftås, Anders Möller, Elena Atanasova, Simone N. Zwicky, Beat Schnüriger, Olga Rutka, Arjun T. Kattakayam, Mushfique Alam, John V. Taylor, Andrei Mihailescu, Eszter T. Karip, Ehtisham Zeb, Adam O’Connor, Goran Pokusevski, Mansoor Khan, Charlotte Florance, Christie Swaminathan, Shameen Jaunoo, Mohammed Sajid, Caoimhe C. Duffy, John Rees, Mark J. Seamon, Niels D. Martin, Ian J. McCurry, Emily A. Vail, Bradford C. Bormann, Daniel C. Cullinane, Jaswin S. Sawhney, Jonathan Dreifus, Forest R. Sheppard, Raul Coimbra, Paul Albini, Sara Edwards

**Affiliations:** 1 Division of Trauma and Emergency Surgery, Orebro University Hospital and School of Medical Sciences, Orebro University, Örebro, Sweden; 2 Division of Traumatology, Surgical Critical Care and Emergency Surgery, Perelman School of Medicine at the University of Pennsylvania, Philadelphia, USA; 3 Corporal Michael Crescenz Veterans Affairs Medical Center, Philadelphia, USA; 4 Helsinki University Hospital HUS Meilahden Tornisairaala, Helsinki, Finland; 5 Servicio de Cirugía General, Unidad de Cirugía de Urgencias, Hospital Universitario Virgen del Rocío, Seville, Spain; 6 Clinical Epidemiology and Biostatistics, School of Medical Sciences, Orebro University, Örebro, Sweden; 7 Royal College of Surgeons in Ireland Medical University, Busaiteen, Bahrain; 8 Center for Perioperative Outcomes Research and Transformation (CPORT), University of Pennsylvania, Philadelphia, USA; 9 Leonard Davis Institute of Health Economics (LDI), University of Pennsylvania, Philadelphia, USA

**Keywords:** Acute appendicitis, COVID-19, Observational cohort, Appendectomy, Outcomes

## Abstract

**Introduction:**

The COVID-19 (SARS-CoV-2) pandemic drove acute care surgeons to pivot from long established practice patterns. Early safety concerns regarding increased postoperative complication risk in those with active COVID infection promoted antibiotic-driven non-operative therapy for select conditions ahead of an evidence-base. Our study assesses whether active or recent SARS-CoV-2 positivity increases hospital length of stay (LOS) or postoperative complications following appendectomy.

**Methods:**

Data were derived from the prospective multi-institutional observational SnapAppy cohort study. This preplanned data analysis assessed consecutive patients aged ≥ 15 years who underwent appendectomy for appendicitis (November 2020–May 2021). Patients were categorized based on SARS-CoV-2 seropositivity: no infection, active infection, and prior infection. Appendectomy method, LOS, and complications were abstracted. The association between SARS-CoV-2 seropositivity and complications was determined using Poisson regression, while the association with LOS was calculated using a quantile regression model.

**Results:**

Appendectomy for acute appendicitis was performed in 4047 patients during the second and third European COVID waves. The majority were SARS-CoV-2 uninfected (3861, 95.4%), while 70 (1.7%) were acutely SARS-CoV-2 positive, and 116 (2.8%) reported prior SARS-CoV-2 infection. After confounder adjustment, there was no statistically significant association between SARS-CoV-2 seropositivity and LOS, any complication, or severe complications.

**Conclusion:**

During sequential SARS-CoV-2 infection waves, neither active nor prior SARS-CoV-2 infection was associated with prolonged hospital LOS or postoperative complication. Despite early concerns regarding postoperative safety and outcome during active SARS-CoV-2 infection, no such association was noted for those with appendicitis who underwent operative management.

## Introduction

Acute appendicitis is one of the most frequent surgical emergencies, and its management is one of the most commonly-performed emergency general surgery procedures [1–4]. While predominantly affecting younger patients [5], appendicitis may occur at any age and exhibits substantial variation in symptoms and severity [1, 6]. Similarly, the etiologies of appendicitis span lymphoid proliferation to appendicolith-associated obstruction and suppuration, to malignant obstruction. Accordingly, a wide range of clinical management approaches are utilized and reflect, in part, clinical equipoise regarding a single optimal management strategy [7].

The initial wave of the SARS-CoV-2 (COVID-19) pandemic raised concerns regarding transmission of infection to operating team members during aerosol generating procedures such as airway control, tracheostomy, endoscopy, or laparoscopy. Concomitantly, safety concerns also surfaced during the COVIDSurg study during the initial phase of the pandemic regarding the advisability of undertaking operative management for patients acutely infected with SARS-CoV-2 [8]. Therefore, acute care surgeons pivoted from established practice patterns to pursue either delayed operative management or, for certain conditions such as appendicitis, non-operative management [10–12]. Importantly, the initial recommendations to pursue non-operative and antibiotic-driven care for those with appendicitis were articulated ahead of an evidence-base documenting enhanced safety. Furthermore, such recommendations may have been predicated upon the anticipation of a short time course for SARS-CoV-2 infection—an assumption that has been well disproved by virus variant evolution and multiple subsequent waves of infection. At the same time, knowledge and experience in caring for patients with SARS-CoV-2 infections improved and global vaccination programs were enacted. Therefore, it is worthwhile examining whether appendicitis patients who underwent appendectomy during later pandemic waves and who were actively infected with SARS-CoV-2, or had been previously infected with SARS-CoV-2, demonstrated prolonged hospitalization related to complications, compared to patients without SARS-CoV-2 infection.

## Methods

This multi-center cohort study adhered to the standardized methodology for snapshot audits [9]. All centers received exemption from informed consent approval from the relevant institutional review board or equivalent as an observational study. All data were anonymized for entry into a secure user-encrypted server running on the Smart-Trial^®^ web application [9]. This study was also conducted in accordance with the Declaration of Helsinki as well as the Strengthening the Reporting of Observational Studies in Epidemiology (STROBE) guidelines [10].

Any site treating emergency general surgery patients was eligible to participate in data collection. No minimum case volume, or center-specific limitations were applied. Data collection was conducted according to a predefined protocol registered with ClinicalTrials.gov (Trial # NCT04365491). The protocol and an invitation to participate was shared by email with registered members of the European Society of Trauma and Emergency Surgery and through national surgical societies. The study enrolled all consecutive patients aged 15 years or older admitted with acute appendicitis during a 3-month window (November 1, 2020–May 28, 2021); enrolled patients were followed for 90 days postoperatively. Data collected included age, sex, American Society of Anesthesiologists (ASA) classification, a history of previous abdominal surgery, ischemic heart disease, insulin-dependent diabetes, congestive heart failure, chronic kidney disease, current smoking status, immunosuppression, the American Association for the Surgery of Trauma (AAST) appendicitis grade, time to surgery from admission, laparoscopic surgery, conversion to open surgery, open surgery, admission white blood cell count and neutrophil percent, admission C-reactive protein concentration, as well as the country where the surgery was performed (Table 1). SARS-CoV-2 seropositivity was determined with a screening PCR or antigen test on admission, based on institutional protocols. The database was closed for analysis on August 31, 2021.

**Table 1:** Demographics, clinical characteristics, and crude outcomes among patients subjected to appendectomy

	No SARS-CoV-2 infection (*N* = 3861)	Active SARS-CoV-2 infection (*N* = 70)	Prior SARS-CoV-2 infection (*N* = 116)	*p*-value
Age, median [IQR]	35 [25–51]	30 [21–43]	37 [28–48]	0.019
Sex, *n* (%)				0.347
Female	1710 (44.3)	36 (51.4)	47 (40.5)	
Male	2145 (55.6)	34 (48.6)	69 (59.5)	
Missing	6 (0.2)	0 (0.0)	0 (0.0)	
Body mass index, mean (SD)	26.1 (± 5.4)	25.4 (± 4.8)	26.5 (± 4.5)	0.486
Missing	933 (24.2)	16 (22.9)	22 (19.0)	
ASA classification, *n* (%)				0.333
1	2333 (60.4)	50 (71.4)	78 (67.2)	
2	1162 (30.1)	17 (24.3)	30 (25.9)	
3	319 (8.3)	2 (2.9)	7 (6.0)	
4	25 (0.6)	0 (0.0)	0 (0.0)	
Missing	22 (0.6)	1 (1.4)	1 (0.9)	
Comorbidities, *n* (%)				
Ischemic heart disease	90 (2.3)	1 (1.4)	2 (1.7)	1.00
Missing	28 (0.7)	0 (0.0)	0 (0.0)	
Congestive heart failure	45 (1.2)	0 (0.0)	1 (0.9)	1.00
Missing	27 (0.7)	0 (0.0)	0 (0.0)	
Insulin-dependent diabetes	73 (1.9)	2 (2.9)	2 (1.7)	0.673
Missing	51 (1.3)	0 (0.0)	1 (0.9)	
Chronic kidney disease	32 (0.8)	0 (0.0)	0 (0.0)	1.00
Missing	20 (0.5)	0 (0.0)	1 (0.9)	
Smoking history, *n* (%)				0.260
Active smoker	422 (10.9)	10 (14.3)	18 (15.5)	
Non-smoker	2555 (66.2)	55 (78.6)	67 (57.8)	
Ex-smoker	259 (6.7)	3 (4.3)	5 (4.3)	
Missing	625 (16.2)	2 (2.9)	26 (22.4)	
Immunosuppression, *n* (%)	77 (2.0)	0 (0.0)	2 (1.7)	0.685
Missing	22 (0.6)	0 (0.0)	0 (0.0)	
Duration of symptoms, *n* (%)				0.949
< 12 h	590 (15.3)	8 (11.4)	18 (15.5)	
12–24 h	1224 (31.7)	24 (34.3)	36 (31.0)	
24–48 h	1011 (26.2)	18 (25.7)	38 (32.8)	
48–72 h	470 (12.2)	9 (12.9)	11 (9.5)	
72–96 h	247 (6.4)	4 (5.7)	6 (5.2)	
> 96 h	302 (7.8)	7 (10.0)	7 (6.0)	
Missing	17 (0.4)	0 (0.0)	0 (0.0)	
Alvarado score, median [IQR]	6.0 [5.0–7.0]	6.0 [4.0–7.0]	6.0 [5.0–7.0]	0.436
Lab results				
WBC (10^9/L), mean (SD)	13.6 (± 5.3)	12.8 (± 4.9)	12.6 (± 3.7)	0.064
Missing	15 (0.4)	0 (0.0)	0 (0.0)	
Neutrophils (%), median [IQR]	81 [73–86]	82 [71–87]	78 [67–86]	0.051
Missing	679 (17.6)	18 (25.7)	24 (20.7)	
CRP (mg/L), median [IQR]	30 [9.0–81]	32 [13–84]	22 [6.0–88]	0.743
Missing	658 (17.0)	8 (11.4)	33 (28.4)	
SIRS, *n* (%)				
Heart rate > 90	763 (19.8)	11 (15.7)	21 (18.1)	0.639
Respiratory rate > 20	111 (2.9)	4 (5.7)	9 (7.8)	0.004
WBC > 12 × 10^9^/L	2,048 (53.0)	34 (48.6)	58 (50.0)	0.613
Missing	2 (0.1)	0 (0.0)	0 (0.0)	
Method of diagnosis, *n* (%)				0.300
Clinical	450 (11.7)	5 (7.1)	12 (10.3)	
Ultrasound	1248 (32.3)	21 (30.0)	46 (39.7)	
CT	2147 (55.6)	44 (62.9)	57 (49.1)	
Missing	16 (0.4)	0 (0.0)	1 (0.9)	
AAST severity, *n* (%)				0.452
Grade 1: acutely inflamed appendix; intact	2058 (53.3)	34 (48.6)	49 (42.2)	
Grade 2: gangrenous appendix; intact	173 (4.5)	4 (5.7)	5 (4.3)	
Grade 3: perforated appendix with local contamination	243 (6.3)	8 (11.4)	6 (5.2)	
Grade 4: perforated appendix with phlegmon/abscess	187 (4.8)	4 (5.7)	4 (3.4)	
Grade 5: perforated appendix with generalized peritonitis	32 (0.8)	2 (2.9)	0 (0.0)	
Missing	1168 (30.3)	18 (25.7)	52 (44.8)	
Time to operation in hours from admission, *n* (%)				0.344
< 6 h	709 (18.4)	10 (14.3)	26 (22.4)	
6–12 h	1387 (35.9)	21 (30.0)	45 (38.8)	
12–24 h	1288 (33.4)	25 (35.7)	31 (26.7)	
> 24 h	458 (11.9)	13 (18.6)	14 (12.1)	
Missing	19 (0.5)	1 (1.4)	0 (0.0)	
Length of procedure in minutes, mean (SD)	62.1 (± 34.1)	64.9 (± 34.0)	58.8 (± 21.8)	0.457
Missing	162 (4.2)	0 (0.0)	1 (0.9)	
Laparoscopic or open				< 0.001
Laparoscopic	3318 (85.9)	51 (72.9)	80 (69.0)	
Laparoscopic converted to open	116 (3.0)	2 (2.9)	3 (2.6)	
Open	370 (9.6)	17 (24.3)	31 (26.7)	
Missing	57 (1.5)	0 (0.0)	2 (1.7)	
Years of surgical experience, median [IQR]	5.0 [3.0–9.0]	5.0 [3.0–10]	4.0 [2.0–7.0]	0.051
Missing	766 (19.8)	7 (10.0)	13 (11.2)	
Preoperative prophylactic antibiotics administered, *n* (%)	3762 (97.4)	68 (97.1)	113 (97.4)	0.850
Postoperative antibiotics administered, *n* (%)	2278 (59.0)	50 (71.4)	72 (62.1)	0.097
Length of stay, median [IQR]	1.9 [1.2–3.2]	2.7 [1.8–5.1]	2.0 [1.5–2.9]	0.002
Missing	123 (3.2)	3 (4.3)	2 (1.7)	
Complications within 30 days, *n* (%)	574 (14.9)	8 (11.4)	13 (11.2)	0.418
None	3280 (85.0)	62 (88.6)	103 (88.8)	0.414
Wound infection	77 (2.0)	2 (2.9)	3 (2.6)	0.573
Wound dehiscence	28 (0.7)	1 (1.4)	1 (0.9)	0.414
Pelvic abscess	130 (3.4)	2 (2.9)	3 (2.6)	1.00
Subphrenic abscess	8 (0.2)	0 (0.0)	0 (0.0)	1.00
Hemorrhage	16 (0.4)	0 (0.0)	0 (0.0)	1.00
Sepsis	29 (0.8)	0 (0.0)	1 (0.9)	0.766
Ileus	109 (2.8)	0 (0.0)	2 (1.7)	0.381
Other complication	292 (7.6)	5 (7.1)	4 (3.4)	0.258
Missing	7 (0.2)	0 (0.0)	0 (0.0)	
Severe complications within 30 days, *n* (%)	110 (2.8)	1 (1.4)	3 (2.6)	0.944
Missing	159 (4.1)	2 (2.9)	1 (0.9)	
Complication severity according to Clavien–Dindo classification, *n* (%)				0.918
None	3285 (85.1)	62 (88.6)	103 (88.8)	
1	146 (3.8)	1 (1.4)	6 (5.2)	
2	161 (4.2)	4 (5.7)	3 (2.6)	
3a	59 (1.5)	0 (0.0)	2 (1.7)	
3b	42 (1.1)	1 (1.4)	1 (0.9)	
4a	2 (0.1)	0 (0.0)	0 (0.0)	
4b	1 (0.0)	0 (0.0)	0 (0.0)	
5	6 (0.2)	0 (0.0)	0 (0.0)	
Missing	159 (4.1)	2 (2.9)	1 (0.9)	
Reoperation, *n* (%)	58 (1.5)	1 (1.4)	2 (1.7)	0.778
Missing	37 (1.0)	1 (1.4)	2 (1.7)	

Temperature is measured in degrees Celsius. Length of stay is measured in days. A severe complication is defined as a Clavien–Dindo classification ≥ 3a

*ASA* American Society of Anesthesiologists, *WBC* white blood cell count, *CRP* C-reactive protein, *SIRS* systemic inflammatory response syndrome, *CT* computed tomography, *AAST* American Association for the Surgery of Trauma

Patients were grouped according to SARS-CoV-2 seropositivity into three sets: uninfected, actively infected, previously infected and recovered; patients whose seropositivity status were unknown or unreported were excluded. Only patients admitted with appendicitis who also underwent appendectomy (laparoscopic or open)—as opposed to non-operative management—formed the dataset explored in this study. Patient demographics, type of operation, hospital length of stay (LOS), as well as complication occurrence, were abstracted from the database for analysis parsed by SARS-CoV-2 seropositivity.

### Statistical analysis

Patient data within each group were summarized as means and standard deviations (SDs) for continuous normally distributed variables, medians, and interquartile ranges (IQRs) for continuous non-normally distributed variables, as well as counts and percentages for categorical variables. An analysis of variance (ANOVA) or a Kruskal-Wallis test was used to assess for differences in continuous variables. A Chi-square test or Fisher’s exact test was used for categorical variables. The primary outcome of interest was any postoperative complication within 30 days, while secondary outcomes of interest were severe complications (Clavien–Dindo classification > 3a) within 30 days and LOS.

The association between a patient’s SARS-CoV-2 seropositivity and complications was analyzed using Poisson regression models with robust standard errors to account for heteroscedasticity [11]. Any complication or severe complications was the dependent variable, while the predictors were the patient’s SARS-CoV-2 seropositivity along with potential confounding variables (Table 2). Results are presented as incident rate ratios (IRRs) and 95% confidence intervals (CIs). The relationship between a patient’s SARS-CoV-2 seropositivity and LOS was explored using a quantile regression model, using LOS as the dependent variable, while SARS-CoV-2 seropositivity and potential confounding variables were included as explanatory variables (Table 3). Results are presented as the change in median LOS along with 95% CIs.

**Table 2:** Incidence rate ratios (IRR) for postoperative complications after an appendectomy, based on SARS-CoV-2 seropositivity

Outcome	IRR (95% CI)	*p*-value
Any complication		
No SARS-CoV-2 infection	Ref.	
Active SARS-CoV-2 infection	0.75 (0.36–1.58)	0.463
Prior SARS-CoV-2 infection	0.87 (0.49–1.55)	0.647
Severe complication		
No SARS-CoV-2 infection	Ref.	
Active SARS-CoV-2 infection	0.83 (0.16–4.41)	0.948
Prior SARS-CoV-2 infection	1.30 (0.41–4.10)	0.662

Poisson regression models with robust standard errors. Multiple imputation with chained equations was used to manage missing values. The models are adjusted for age, sex, American Society of Anesthesiologists classification, a history of previous abdominal surgery, ischemic heart disease, insulin-dependent diabetes, congestive heart failure, chronic renal disease, current smoking status, immunosuppression, the American Association for the Surgery of Trauma appendicitis grade, time to surgery from admission, laparoscopic surgery, conversion to open surgery, open surgery, white blood cell count on admission, neutrophil percent on admission, C-reactive protein level on admission, as well as the country where the surgery was performed

**Table 3:** Change in median length of stay after an appendectomy, based on SARS-CoV-2 seropositivity

	Change in median length of stay (95% CI)	*p*-value
No SARS-CoV-2 infection	Ref.	
Active SARS-CoV-2 infection	0.21 (− 0.23 to 0.65)	0.353
Prior SARS-CoV-2 infection	0.12 (− 0.03 to 0.26)	0.114

Length of stay is measured in days. Quantile regression model. Multiple imputation with chained equations was used to manage missing values. The model was adjusted for age, sex, American Society of Anesthesiologists classification, previous abdominal surgery, ischemic heart disease, insulin dependent diabetes, congestive heart failure, chronic renal disease, smoking history, immunosuppression, American Association for the Surgery of Trauma appendicitis grade, time to surgery from admission, laparoscopic surgery, conversion to open surgery, open surgery, white blood cell count on admission, neutrophil percent on admission, C-reactive protein level on admission, as well as the country where the surgery was performed

A two-tailed *p* value < 0.05 was considered statistically significant in all analyses. Missing data were managed using multiple imputation by chained equations. Logistic regression models were used for binary variables, and Bayesian polytomous regression was used for nominal variables. Proportional odds models were used for ordinal variables. Analyses were performed using statistical software R (R Foundation for Statistical Computing, Vienna, Austria) with the tidyverse, mice, lubridate, readxl, writexl, robustbase, and quantreg packages [12].

## Results

Four thousand forty seven consecutive patients from 71 centers in 14 countries were included in the dataset; these countries included Bahrain, Estonia, Finland, Iran, Ireland, Israel, Italy, Portugal, Romania, Spain, Sweden, Switzerland, the UK, and the USA [13]. The majority were SARS-CoV-2 uninfected (3861, 95.4%), while 70 (1.7%) were acutely SARS-CoV-2 positive, and 116 (2.8%) reported prior SARS-CoV-2 infection. Patients with an active SARS-CoV-2 infection were younger compared to patients with and without prior infection [median (IQR) 30 (21–43) years vs 37 (28–48) years in those with a history of prior infection and 35 (25–51) years in those never infected, *p* = 0.019]. There were no statistically significant between-cohort differences in body mass index, ASA classification, comorbidities, smoking history, or admission laboratory data. However, the proportion of patients with a respiratory rate above 20 was significantly higher among patients with active and previous SARS-CoV-2 infections compared to patients who had never had SARS-CoV-2 (5.7% and 7.8% vs 2.9%, respectively, *p* = 0.004) (Table 1).

The prevalence of perforation and the time to surgery from admission were similar across groups. Patients with active and prior SARS-CoV-2 infections were significantly more likely to undergo an open surgical procedure (24.3% and 26.7% vs 9.6%, respectively, *p* < 0.001). Relatedly, procedure duration and the crude rate of any or severe complications were also similar across groups. Patients with active SARS-CoV-2 infection demonstrated a longer crude LOS compared to patients without prior SARS-CoV-2 infection (2.7 days vs 1.9 days, *p* = 0.002) (Table 1). No associations between SARS-CoV-2 seropositivity and LOS, any complication, or severe complication were identified after adjusting for confounders in the regression analyses (Tables 2 and 3).

## Discussion

The treatment of patients with acute surgical emergencies in the context of the COVID-19 pandemic has been challenging throughout different phases of the pandemic—from early knowledge gaps and resource-exhaustion, through concern for excess postoperative morbidity and potential aerosolization of viral particles through laparoscopy, to cancellation of elective surgery and deviation from usual practice patterns, the introduction of population vaccination, and most recently, phased reintroduction of scheduled surgical services [14–16].

The ‘evidence-to-practice gap’ between guideline-based recommendations and widespread adoption and implementation is well chronicled in the surgical literature and a burgeoning focus of implementation science [17]. Under usual conditions, there are several known barriers to recommendation implementation, including care inertia, lack of knowledge of new recommendations, resource limitation, as well as disagreement with guideline recommendations [17, 18]. In stark comparison, the recent SARS-CoV-2 global pandemic engendered rapid creation, adoption and implementation of recommendations ahead of mature large-data evidence [19]. Concerns regarding staff safety as well as untoward patient outcomes following operative procedures performed during active SARS-CoV-2 infection shuttered elective and semi-elective procedures [20–22]. Multiple medical professional organizations, including the European Society for Trauma and Emergency Surgery (ESTES), rapidly crafted guidelines and statement. These supported delaying operative therapy, and prioritizing non-operative approaches, during acute SARS-CoV-2 infection as well as recommending the avoidance of laparoscopic interventions due to the potential risk of the uncontrolled release of pressurized gas, which could result in the infection of surgical staff [23–26]. Accordingly, urgent procedures, including appendectomy for acute appendicitis, were diverted along a non-operative pathway. As a result, the recommendation for non-operative appendicitis management was readily embraced by surgeons who would previously have pursued routine operative management [27]. Only emergency operations such as those for injury, or life-saving organ transplantation, were undertaken during the early phase of the pandemic. There was a clear need for data to inform practice.

Multinational collaborations rapidly arose to assess outcomes of different therapeutics for those with acute SARS-CoV-2 infection. Some early therapies, such as glucocorticoid administration, remdesivir, monoclonal antibody rescue, and prone position therapy were found to be beneficial when large datasets were interrogated [28, 29]. Other practices driven by early observations, such as routine therapeutic anticoagulation or early invasive mechanical ventilation, have been intensively investigated, determined to be lacking an evidence base, and abandoned as part of routine care [30–32]. Our data aligned with the latter studies in that we identified *no untoward consequences* for those with active SARS-CoV-2 infection who underwent appendectomy with active infection compared to those without infection, as well as those who recovered from a prior infection [33]. Since those who undergo appendectomy regularly demonstrate excellent outcomes, any deleterious impact of active SARS-CoV-2 infection is anticipated to be readily recognizable, even in a small cohort. It was also apparent that the recommendations regarding operative technique had an effect, given that the proportion of patients who underwent an open surgery, among those that had an active or prior SARS-CoV-2 infection, was nearly triple the proportion observed in uninfected patients.

The outcomes from this study span 90 days, a sufficient time frame to capture delayed events including hospital readmission for operative domain or pulmonary system failures; these adverse events were not observed. Importantly, our data are different from observations made early in the pandemic. One reason for such differences may be viral evolution, a process that has been repeatedly observed with different variants with some demonstrating enhanced infectivity but less virulence, and vice versa [34, 35]. Other explanations include changes in the vulnerable patient population and enhanced acute care paradigms—a key aspect as our data were collected during the European second and sometimes third wave (delta variant dominant periods). It is also possible that patient selection informed which patients were deemed suitable for operation, potentially selecting a less physiologically encumbered group. While that is possible, we did not observe differences between groups with regard to ASA score or overall comorbidity burden. It is also possible that intra-operative pulmonary management shifted to routinely utilizing PEEP in the wake of additional understanding of acute SARS-CoV-2 infection. Such an approach may have reduced atelectasis and supported pulmonary flow particularly during abdominal insufflation and may have contributed to the observed lung-related outcomes. This is a supposition that is plausible, and one that is not investigable from the current database—a shortcoming of using a database with pre-specified fields as is typical for snapshot audits and other prospective analyses [9].

Our prospective time-bound multi-center observational cohort study allowed for a comprehensive defined dataset to be gathered in line with pre-publication, open-access protocols filed with clinical trial repositories. Based on the pre-specified data fields, we did not capture all comorbidities, nor how patients were selected for non-operative versus operative management. Moreover, our data is limited to those with acute appendicitis and may not be applicable to those who required longer or more complex operative procedures, nor those who may require postoperative invasive mechanical ventilation. Similarly, we did not capture SARS-CoV-2 related therapy such as steroids, remdesivir, or the use of non-invasive ventilation or prone position therapy. Relatedly, we did not capture whether patients were ill from their SARS-CoV-2 infection, or asymptomatically infected as existing recommendations addressed such infection in a binary fashion. We were instead interested in assessing outcome across multiple centers for those who underwent operation regardless of how their acute SARS-CoV-2 infection was managed. Indeed, current management leverages some different than what was used during our study period. Additionally, our results present the median effects observed in the study population. We can therefore not eliminate the risk that a particular subgroup, for example frail or elderly patients, might exhibit an increased risk when undergoing surgical management for acute appendicitis with a concomitant SARS-CoV-2 infection. Nevertheless, owing to the age distribution of appendicitis patients, this cohort is relatively small with only 3.5% of patients in this sample being older than 75 and none of them having an active SARS-CoV-2 infection on admission [5]. Furthermore, the usual caveats applicable to observational studies also apply, such as the risk of residual confounding, selection bias, and limitations on causal inference.

## Conclusions

The current study failed to detect any association between SARS-CoV-2 infection status and post-appendectomy complications or hospital length of stay. This provides evidence that the most common management approach for acute appendicitis—appendectomy—may be safely performed in patients who present with acute or recently recovered SARS-CoV-2 infection.

## Data Availability

All data and codes are available for retrieval on reasonable request.

## References

[1] Humes DJ, Simpson J (2006). Acute appendicitis. BMJ.

[2] van Rossem CC, Bolmers MDM, Schreinemacher MHF, van Geloven AAW, Bemelman WA, Snapshot Appendicitis Collaborative Study Group (2016). Prospective nationwide outcome audit of surgery for suspected acute appendicitis. Br J Surg.

[3] Stewart B, Khanduri P, McCord C, Ohene-Yeboah M, Uranues S, Vega Rivera F (2014). Global disease burden of conditions requiring emergency surgery. Br J Surg.

[4] Bhangu A, RIFT Study Group on behalf of the West Midlands Research Collaborative (2020). Evaluation of appendicitis risk prediction models in adults with suspected appendicitis. Br J Surg.

[5] Viniol A, Keunecke C, Biroga T, Stadje R, Dornieden K, Bösner S (2014). Studies of the symptom abdominal pain—a systematic review and meta-analysis. Fam Pract.

[6] Di Saverio S, Podda M, De Simone B, Ceresoli M, Augustin G, Gori A (2020). Diagnosis and treatment of acute appendicitis: 2020 update of the WSES Jerusalem guidelines. World J Emerg Surg.

[7] National Surgical Research Collaborative (2013). Multicentre observational study of performance variation in provision and outcome of emergency appendicectomy. Br J Surg.

[8] Glasbey JC, Nepogodiev D, Simoes JFF, Omar O, Li E, Venn ML (2021). Elective cancer surgery in COVID-19-free surgical pathways during the SARS-CoV-2 pandemic: an international, multicentre, comparative cohort study. J Clin Oncol.

[9] Bass GA, Kaplan LJ, Ryan ÉJ, Cao Y, Lane-Fall M, Duffy CC, et al. The snapshot audit methodology—design, implementation and analysis of prospective observational cohort studies in surgery. Eur J Trauma Emerg Surg. 2022. 10.1007/s00068-022-02045-3 . 10.1007/s00068-022-02045-3 PMC10606835 35840703

[10] WMA—The World Medical Association-WMA Declaration of Helsinki—Ethical principles for medical research involving human subjects [Internet]. [cited 2020 Sep 21]. https://www.wma.net/policies-post/wma-declaration-of-helsinki-ethical-principles-for-medical-research-involving-human-subjects/. Accessed 22 June 2022.

[11] Petersen M (2009). Estimating Standard errors in finance panel data sets: comparing approaches. Rev Financ Stud.

[12] R Development Core Team. R: a language and environment for statistical computing [Internet]. Vienna: R Foundation for Statistical Computing; 2008. http://www.R-project.org/. Accessed 22 June 2022.

[13] ESTES SnapAppy Group. Practice pattern variability and outcomes following appendectomy for acute appendicitis: an ESTES’ snapshot audit’ of international practice. Eur J Trauma Emerg Surg. 2022 (**under review**).

[14] Pagel C (2022). The covid waves continue to come. BMJ.

[15] Gutman R. Another COVID wave is looming [Internet]. The Atlantic. 2022 [cited 2022 Jun 22]. https://www.theatlantic.com/health/archive/2022/03/omicron-subvariant-new-covid-wave/627094/. Accessed 22 June 2022.

[16] Prater E. A new wave of COVID is coming, and America doesn’t seem to care [Internet]. Fortune. [cited 2022 Jun 22]. https://fortune.com/2022/04/09/new-covid-wave-return-to-office-fauci-omicron-subvariant/. Accessed 22 June 2022.

[17] Lane-Fall MB, Curran GM, Beidas RS (2019). Scoping implementation science for the beginner: locating yourself on the “subway line” of translational research. BMC Med Res Methodol.

[18] Lane-Fall MB, Cobb BT, Cené CW, Beidas RS (2018). Implementation science in perioperative care. Anesthesiol Clin.

[19] Kearsley R, Duffy CC (2020). The COVID-19 information pandemic: how have we managed the surge?. Anaesthesia.

[20] COVIDSurg Collaborative, GlobalSurg Collaborative. Timing of surgery following SARS-CoV-2 infection: an international prospective cohort study. Anaesthesia. 2021;76(6):748–58. 10.1111/anae.15458 PMC8206995 33690889

[21] COVIDSurg Collaborative, GlobalSurg Collaborative (2021). Effects of pre-operative isolation on postoperative pulmonary complications after elective surgery: an international prospective cohort study. Anaesthesia.

[22] COVIDSurg Collaborative, GlobalSurg Collaborative (2022). SARS-CoV-2 infection and venous thromboembolism after surgery: an international prospective cohort study. Anaesthesia.

[23] Coimbra R, Edwards S, Kurihara H, Bass GA, Balogh ZJ, Tilsed J (2020). European Society of Trauma and Emergency Surgery (ESTES) recommendations for trauma and emergency surgery preparation during times of COVID-19 infection. Eur J Trauma Emerg Surg.

[24] COVID-19 Guidelines for Triage of Emergency General Surgery Patients [Internet]. American College of Surgeons. [cited 2022 Jun 22]. https://www.facs.org/for-medical-professionals/covid-19/clinical-guidance/elective-case/emergency-surgery/. Accessed 22 June 2022.

[25] Updated Intercollegiate General Surgery Guidance on COVID-19 [Internet]. Royal College of Surgeons. [cited 2022 Jun 22]. https://www.rcseng.ac.uk/coronavirus/joint-guidance-for-surgeons-v2/. Accessed 22 June 2022.

[26] Updated General Surgery Guidance on COVID-19—30th May 2020 [Internet]. The Royal College of Surgeons of Edinburgh. [cited 2022 Jun 22]. https://www.rcsed.ac.uk/news-public-affairs/news/2020/june/updated-general-surgery-guidance-on-covid-19-30th-may-2020. Accessed 22 June 2022.

[27] Köhler F, Müller S, Hendricks A, Kastner C, Reese L, Boerner K (2021). Changes in appendicitis treatment during the COVID-19 pandemic—a systematic review and meta-analysis. Int J Surg.

[28] Dequin PF, Heming N, Meziani F, Plantefève G, Voiriot G, Badié J (2020). Effect of hydrocortisone on 21-day mortality or respiratory support among critically ill patients with COVID-19: a randomized clinical trial. JAMA.

[29] Mehta A, Bansal M, Vallabhajosyula S. In COVID-19 acute hypoxemic respiratory failure, awake prone positioning vs. the supine position reduces intubations. Ann Intern Med. 2022;175(7):JC81. 10.7326/J22-0050 35785529

[30] Kavecansky J, Dusendang JR, Tavakoli J, Schmittdiel J, Ho G, Loyles J (2020). Association of anticoagulant use with COVID-19 diagnosis. Blood.

[31] Patel NG, Bhasin A, Feinglass JM, Angarone MP, Cohen ER, Barsuk JH (2021). Mortality, critical illness, and mechanical ventilation among hospitalized patients with COVID-19 on therapeutic anticoagulants. Thromb Update.

[32] Hohmann F, Wedekind L, Grundeis F, Dickel S, Frank J, Golinski M (2022). Early spontaneous breathing for acute respiratory distress syndrome in individuals with COVID-19. Cochrane Database Syst Rev.

[33] Huamán Egoávil E, LaGrone L, Ugarte Oscco R, Endo Ramos S, Diaz Baltazar A, Vergel CC (2021). SARS-CoV-2 infection is not associated with a higher rate of post-operative complications in adult appendectomy patients in Peru: cross-sectional study. Ann Med Surg (Lond).

[34] Musa-Booth TO, Adegboro B, Medugu N (2022). Evolution of SARS-CoV-2 variants: a mini-review. Afr J Clin Exp Microbiol.

[35] Szanyi J, Wilson T, Howe S, Zeng J, Andrabi H, Blakely T. An integrated epidemiologic and economic model to assess optimal COVID-19 pandemic policy [Internet]. medRxiv; 2022 [cited 2022 Aug 14]. p. 2022.08.01.22278262. https://www.medrxiv.org/content/10.1101/2022.08.01.22278262v2

